# Effect of a Multispecies Probiotic Mixture on the Growth and Incidence of Diarrhea, Immune Function, and Fecal Microbiota of Pre-weaning Dairy Calves

**DOI:** 10.3389/fmicb.2021.681014

**Published:** 2021-07-14

**Authors:** Yanyan Wu, Lili Wang, Ruiqing Luo, Hongli Chen, Cunxi Nie, Junli Niu, Cheng Chen, Yongping Xu, Xiaoyu Li, Wenjun Zhang

**Affiliations:** ^1^College of Animal Science and Technology, Shihezi University, Shihezi, China; ^2^School of Bioengineering, Dalian University of Technology, Dalian, China; ^3^Xinjiang Tianshan Junken Animal Husbandry Co., Ltd., Shihezi, China

**Keywords:** multispecies probiotic, growth, diarrhea, microbiota, calves

## Abstract

The effects of different doses of a multispecies probiotic (MSP) mixture on growth performance, the incidence of diarrhea rate and immune function, and fecal microbial diversity and structure were evaluated in pre-weaning Holstein dairy calves at WK2, WK4, WK6, and WK8. Forty Chinese Holstein female newborn calves were randomly assigned to four treatments with 10 calves in each group, C (control group), T1 (0.5 g MSP/calf/day, T2 (1 g MSP/calf/day), and T3 (2 g MSP/calf/day) groups. The experimental period was 56 days. Feed intake and health scoring were recorded every day until the end of the experiment. Fecal contents and blood samples were sampled at WK2, WK4, WK6, and WK8. Growth performance, incidence of diarrhea, and total serum concentrations (IgA, IgG, and IgM) were analyzed. Bacterial 16S rRNA and fungal ITS genes were high-throughput sequenced for fecal microbiota. The relationships among the populations of the principal fecal microbiota at WK2 and the growth performance or serum immunoglobulin concentrations were analyzed using Pearson’s rank correlation coefficients. The MSP supplementation reduced the incidence of diarrhea in the first 4 weeks of life, and serum IgA, IgG, and IgM concentrations increased between WK2 and WK8 in the T3 group. There was an increase in growth performance and reduction in the incidence of diarrhea until WK4 after birth in T3 group, compared with the control, T1, and T2 groups. The results of fecal microbiota analysis showed that *Firmicutes* and *Bacteroides* were the predominant phyla, with *Blautia*, *Ruminococcaceae_UCG-005*, *norank_f__Muribaculaceae*, *Bacteroides*, *Subdoligranulum*, and *Bifidobacterium* being the dominant genera in calf feces. *Aspergillus*, *Thermomyces*, and *Saccharomyce*s were the predominant fungal phyla. Compared with the control, in T1 and T2 groups, the MSP supplementation reduced the relative abundance of *Bacteroidete*s and increased the relative abundance of *Bifidobacterium*, *Lactobacillus*, *Collinsella*, and *Saccharomyces* at WK2 in group T3. Thus, the fecal microbial composition and diversity was significantly affected by the MSP mixture during the first 2 weeks of the calves’ life. MSP mixtures reduced the incidence of diarrhea in pre-weaning calves (during the first 4 weeks of life). There was a significant improvement in growth performance, reduction in calf diarrhea, balance in the fecal microbiota, and an overall improvement in serum immunity, compared with the control group. We, therefore, recommend adding 2 g/day of multispecies probiotic mixture supplementation in diets of dairy calves during their first 4 weeks of life before weaning.

## Introduction

Neonatal diarrhea occurs frequently in dairy calves all over the world, causing huge economic and productivity losses that undermine healthy and sustainable development of animal husbandry (El-Seedy et al., 2016). Moreover, even if calves recover from the diarrhea, their subsequent growth and development are hindered, which later affects their productivity in adulthood (Heinrichs and Heinrichs, 2011). Generally, feed supplementation could reduce the incidence of diarrhea and improve the health of calves. Therefore, it is very important to determine the application of effective antidiarrheal agents (Wang et al., 2018) in dairy farming since the European Union (Casewell et al., 2003) and China (No. 194, 2020) prohibited the use of antimicrobial growth promoters.

In 2014, the International Association of Probiotics and Prebiotic Sciences (ISAPP) emphasized the importance of probiotics in improving the survivability of animals (Markowiak and Śliżewska, 2018). Probiotics are defined as “living organisms that bring health benefits to the host at an appropriate dose” (Hill et al., 2014). Multispecies probiotics (MSPs) were more effective than single-strain probiotics, especially in treating antibiotic-associated diarrhea in children (Ki et al., 2012; Łukasik and Szajewska, 2018), improving animal growth performance (Renaud et al., 2019), resisting bacterial infection (Perdigon et al., 1990; Lema et al., 2001), weight gain after stimulation post-enteritis (Renaud et al., 2019), and improving intestinal microbiota (Hod et al., 2018; Biagioli et al., 2019).

Multispecies probiotics [*Lactobacillus acidophilus* (McFarland et al., 2018; Łukasik and Szajewska, 2018), *Bacillus subtilis* (Rui and Ma, 2020), *Saccharomyces cerevisiae* (Thévenot et al., 2015)] have achieved certain results in human application, and there are similar reports in animals. Studies have found that *Lactobacillus acidophilus* (Sharma et al., 2018), *Bacillus subtilis* (Sun et al., 2010; Zhang et al., 2017), and *Saccharomyces cerevisiae* (Fomenky et al., 2018; Villot et al., 2019) can improve calf growth performance by improving immune function and balancing the structure of intestinal microbiota. The objectives of this study are: (1) To evaluate whether MSP supplementation can reduce the incidence of diarrhea in pre-weaning calves while improving the growth performance. (2) To evaluate whether the MSP supplementation can improve serum immunity (IgA, IgG, and IgM) in pre-weaning calves. (3) To evaluate whether MSP supplementation can affect the diversity and composition of the fecal microbiota of pre-weaning calves.

## Materials and Methods

This study has been approved by the ethics committee of the College of Animal Science and Technology, Shihezi University (No. A2019-155-01).

### Preparation of the Multispecies Probiotics Mixture

Probiotic strains of *Lactobacillus acidophilus S5* (Wu, 2013), *Bacillus subtilis* No. Bzg988118 (Bao, 2013), and *Saccharomyces cerevisiae SHZ2017* were provided by the Biological Feed Laboratory of the College of Animal Science and Technology, Shihezi University, China. *In vitro* analyses revealed that all three strains have the potential benefits of probiotics, inhibiting the growth of Gram-positive and Gram-negative pathogens (i.e., *Escherichia coli* K99, *Salmonella*, and *Staphylococcus aureus*), resist low pH and bovine bile salts, and tolerance to artificial gastrointestinal environment (Wu et al., 2021).

Each of the three strains were cultured, respectively, in de Man, Rogosa, and Sharpe medium (MRS), yeast peptone dextrose (YPD), and Luria–Bertani (LB) medium (purchased from Qingdao Gaokeyuan Haibo Biotechnology Co., Ltd., Qingdao, China), where *L. acidophilus* was anaerobically cultured at 37°C for 20 h, while *S. cerevisiae* and *B. subtilis* were cultured on a shaker at 37°C for 20 h, as described by Dong et al. (2013) after cultivation. One liter of the bacterial culture enrichment was centrifuged at 4,000 rpm for 3 min to remove the bacterial supernatant. The precipitation was washed with 60 ml of sterile PBS buffer including 5% glycerol and 20% skim milk powder (Shu et al., 2015), then mixed with 0.25 kg bran and freeze dried. The ratio of the three strain probiotics complex was 3:3:1, representing *L. acidophilus*, *B. subtilis*, and *S. cerevisiae* fermentum based on previous research (Wu et al., 2021).

### Animals and Diet

Forty Chinese Holstein female calves (age = 6 ± 3 days, BW = 40.86 ± 2.65 kg) were selected and randomly assigned into four treatment groups with 10 calves per group. All the calves were removed from their dams immediately after birth and housed in individual pens (1.8 × 1.4 × 1.2 m), which were bedded with straw and had iron fences to avoid cross-contamination for the entire length of the experiment (December 2019–February 2020). Calves were fed 4 L of colostrum (pasteurized at 60°C for 1 h) from a bottle within 1 h of birth. The calves were fed twice with milk in two equal-volume plastic buckets daily, at 0700 and 1800 h. On day 5, the volume of feed was increased to 6 L/day (3 L/meal) of milk, which was produced in the same farm and pasteurized at 60°C for 1 h. On day 6, the volume of feed was increased again to 7 L/day (3.5 L/meal), and finally, on day 7 to 53, the volume of feed was 8 L/day (4 L/meal), which was gradually reduced to zero by 1 L/day until weaning at day 61. Starter concentrates were provided by Xinjiang Urumqi Zhengda Feed Co., Ltd. (Urumqi, China) and was fed to the calves from day 4. All calves received the same colostrum and milk. The MSP (1 g MSP contains *L. acidophilus* 3 × 10^9^ CFU, *B. subtilis* 3 × 10^9^ CFU, and *S. cerevisiae* 1 × 10^9^ CFU) was prepared by the Biological Feed Laboratory of the College of Animal Science and Technology, Shihezi University (Wu et al., 2021). The ingredients and chemical composition of the starter concentrates are shown in Table 1.

**TABLE 1:** Ingredient composition and nutrient levels of starter (DM basis).

**Item**	**Value**
Ingredient, g/kg of DM	
Corn	55.20
Soybean meal^1^	18.50
Corn gluten meal	10.00
DGGS^2^	13.00
Limestone	1.80
NaCl	0.50
Premix^3^	1.00
Total	100.00
Chemical analysis	
DM, g/kg	87.33
CP, g/kg	19.92
Ether extract, g/kg	4.64
ADF, g/kg	6.02
NDF, g/kg	16.53
Ash, g/kg	5.38
Calcium	1.15
Phosphorus	0.58

*^1^Soybean meal: 89.1% DM and 42.6% CP.^2^DDGS, distiller’s dried grains with solubles are the nutrient-rich co-product of dry-milled ethanol production.^3^Premix provides the following per kg of the starter diet: VA 15,000 IU, VD 5,000 IU, VE 50 mg, Fe 90 mg, Cu 12.5 mg, Mn 30 mg, Zn 90 mg, Se 0.3 mg, I 1.0 mg, and Co 0.5 mg.*

### Experimental Design and Sample Collection

The MSP was provided in the form of freeze-dried powder and was mixed with fresh cow’s milk. The control calves were fed with starter and milk that was not supplemented with MSP, while the calves in the treatment (T) groups received MSP: T1 at 0.5 g/calf/day, T2 at 1 g/calf/day, and T3 at 2 g/calf/day. No additives were fed to the control group. Before the start of the 8-week trial experiment, animals were individually checked for signs of disease, injury, and dehydration, and those that were initially deemed unhealthy were not included as part of the 40 calves used. The trial lasted for 56 days, during which all the animals had free access to the same fresh water and starter concentrate. This study was conducted between December 2019 and February 2020 at Shurui Farm, Tianshan Co., Shihezi, China.

Blood samples were obtained from six calves per group by jugular vein puncture using 10-ml of gel vacuum tubes on the morning of WK2, WK4, WK6, and WK8. Samples were centrifuged at 3,000 × *g* for 15 min at 4°C using a high-speed refrigerated centrifuge Eppendorf 5810R (Eppendorf AG, Hamburg, Germany). Separated serum was stored at −20°C for subsequent total serum IgA, IgG, and IgM measurements.

Fecal and blood samples were collected from the same six calves per group at WK2, WK4, WK6, and WK8 using sterile gloves after feeding for 3 h. Before collecting the samples, centrifuge tubes (Corning, NY, United States) were sterilized in an autoclave and then used to collect the fresh feces. A rectal palpation method was used to collect a stool sample (20 g) directly from the rectum. During sampling, the sample was stored in a 15-ml cryovial on ice with about 0.2 g of feces being collected into each 2-ml tube. A total of four tubes were filled for six animals from each group. These fecal samples were snap-frozen in liquid nitrogen and stored at −80°C for later analyses.

### Analysis of Growth Performance and the Incidence of Diarrhea

The average daily gain (ADG) was calculated by weighing the calves on days 1 and 56. The DMI of milk and starter was also recorded throughout the trial. The starter was sampled for analyses of DM content (AOAC International, 2005; method 930.15), CP (AOAC International, 2000; method 976.05), and ether extract (AOAC International, 2003; method 4.5.05) using standard procedures of the AOAC International. The NDF and ADF contents were determined as described by Van Soest et al. (1991).

A standard health scoring system (Renaud et al., 2018) was used for the fecal scores every morning at 1000 h. In short, fecal consistency was scored on the following scale: 0 = normal, 1 = half-shaped and pasty, 2 = loose but staying on the mat, and 3 = watery, sieve through the mat. A case of diarrhea is defined when the fecal score is at least 2 (Lesmeister et al., 2004). The following formula was used to calculate the incidence of diarrhea in each group.

Incidence of diarrhea% = calves with diarrhea in each group × diarrhea days/(total calves in each group × experimental days) × 100%.

### Analysis of Total Serum IgA, IgG, and IgM Concentrations by ELISA

The total serum IgA, IgG, and IgM antibody concentrations were determined using bovine ELISA kits purchased from NanJing JianCheng Bioengineering Institute (Nanjing, China). All tests were run according to the manufacturer’s protocols.

### Bacterial 16S rRNA Gene and Fungal ITS Gene High-Throughput Sequencing

Genomic DNA from the microbial community in the fecal samples was extracted by using the E.Z.N.A.^®^ soil DNA kit (Omega Bio-Tek, Norcross, GA, United States) according to the manufacturer’s protocol. The extracted DNA was checked on a 1% agarose gel, and DNA concentration and purity were determined using a NanoDrop 2000 UV-vis spectrophotometer (Thermo Scientific, Wilmington, NC, United States).

The primer sequences for the amplification of bacterial V3–V4 region are: F: 5′-ACTCCTACGGGAGGCAGCAG-3′ and R: 5′-GGACTACHVGGGTWTCTAAT-3′ (Chang et al., 2020). PCR conditions were as follows: 95°C for 3 min; 35 cycles at 95°C for 30 s, 55°C for 30 s, and 72°C for 45 s, followed by a hold at 72°C for 10 min. The primer sequences for the amplification of fungal ITS1F–ITS2R region amplification are F: 5′-CTTGGTCATTTAGAGGAAGTAA-3′ and R: 5′-GCTGCGTTCTTCATCGATGC-3′ (Hoffmann, 2013). PCR conditions were as follows: 95°C for 3 min; 27 cycles at 95°C for 30 s, 55°C for 30 s, and 72°C for 45 s; followed by a hold at 72°C for 10 min. The sequences were submitted to GenBank, and the accession number obtained is BioProject ID: PRJNA692054.

### Illumina MiSeq Sequencing

Purified amplicons were pooled in equimolar and paired-end sequenced on an Illumina MiSeq PE300 platform/NovaSeq PE250 platform (Illumina, San Diego, CA, United States) following the standard protocols by Majorbio Bio-Pharm Technology Co., Ltd. (Shanghai, China).

### Processing of Sequence Data

The key steps of sequencing data analysis are briefly described here. To get clean reads, the raw data had to be preprocessed to eliminate adapter contamination and low-quality data. Sequence data was quality filtered using FastQC version 0.20.0 (Chen et al., 2018) and merged using FLASH version 1.2.7 (Magoč and Salzberg, 2011). The bacterial and fungal tags were clustered into operational taxonomic units (OTUs) by the QIIME (v7.1) software based on 97% sequence similarity using the UPARSE script (Edgar, 2013). Representative OTU sequences of fungi were classified using the ribosomal database project (RDP) classifier v.2.2 based on the UNITE database (Abarenkov et al., 2010). Then Majorbio cloud software was used to perform several key analyses, including α diversity (including observed species, Shannon, Simpson index, Ace, and Chao1), species composition (heatmap, bar), beta diversity (beta), diversity analysis (including PCA), linear discriminant analysis effect size (LEfSe), and association analysis.

### Statistical Analysis

The Durbin–Watson test was used to check the randomness of the initial and final BW data to test that the randomization had been effective. A chi-squared contingency test was used to compare the effect of MSP on the prevalence of diarrhea. The growth performance data was analyzed using a one-way ANOVA in the MIXED procedure of SAS 9.4. The serum immunoglobulin concentration and fecal microbial data were analyzed on the basis of repeated measurements, and a compound symmetry variance and covariance structure using the GLIMMIX procedure of SAS 9.4. The repeated measures model contained fixed effects of treatment, day, and the interaction of treatment and day, and the random effect of calf identity. The data are presented as the least squares mean and standard error of the mean. Differences between the treatment groups were identified using Tukey’s multiple range test. A *p* ≤ 0.05 was accepted as statistically significant, and *p*-values between 0.05 and 0.10 were considered to represent a statistical trend. The relationships between the populations of the principal fecal microbes on day 14 and the growth performance or serum immunoglobulin concentrations were analyzed using Pearson’s rank correlation coefficients.

## Results

### Performance and Incidence of Diarrhea

No differences were observed in the initial or final BW of the calves in the four groups. Supplementation with MSP in group T2 significantly increased the ADG of calves when compared with the control group (*p* < 0.05; Table 2). No differences were observed in the DMI of starter or in the feed efficiency between the four groups. However, total feed intake by groups T1, T2, and T3 were much higher than that of the control group (*p* < 0.05). There was a slight reduction in the incidence of diarrhea in calves in group T3 when compared with the control group at WK2 (12.14 vs. 25.11; *p* = 0.02). In addition, supplementing with MSP reduced the incidence of diarrhea during the first 4 weeks of life in group T3 compared with the C, T1, and T2 groups (*p* = 0.02; *p* = 0.04).

**TABLE 2:** The growth performance and incidence of diarrhea in Holstein dairy calves fed with different doses of multispecies probiotics.

**Items**	**Treatment (Trt)^1^**				**SEM**	***p*-Value**
	**C**	**T1**	**T2**	**T3**		**Trt**
Initial BW, kg	41.12	42.22	41.44	41.16	2.65	0.28
Final BW, kg	82.96	87.16	89.12	86.98	2.99	0.10
ADG, g/d	664.29^b^	815.71^ab^	875^a^	720^b^	27.69	0.03
Starter intake, g of DM/d	24.20^b^	29.58^a^	28.66^a^	30.82^a^	0.67	0.08
Total feed intake, g of DM/d	1,241.61^b^	1,306.44^a^	1,295.35^a^	1,321.44^a^	8.1	<0.01
Feed efficiency, g of DMI/g of gain	1.34	1.25	1.34	1.44	0.03	0.61
Incidence of diarrhea (d 1 to d 14), %	25.11	20	20	12.14	0.049	0.02
Incidence of diarrhea (d 15 to d 28), %	1.14	1.13	1.85	–	0.041	0.04
Incidence of diarrhea (d 29 to d 42), %	–	1.13	–	–	0.028	0.587
Incidence of diarrhea (d 43 to d 56), %	–	–	–	–	NS	NS

*^*a*,*b*^The values in the same row with different superscripts are significantly different (*p* < 0.05), while values with the same or no superscripts mean no significant difference (*p* > 0.05).^1^Treatments: C, no supplementation (control); T1 = 0.5 g/calf/day; T2 = 1 g/calf/day; T3 = 2 g/calf/day.ADG, average daily gain; SEM, standard error of mean.*

### Total Serum IgA, IgG, and IgM Concentrations

The total serum IgA and IgM concentrations in group T3 were significantly higher than those in the control group between WK2 to WK8 (*P* < 0.05; Table 3). The total serum IgG concentration in group T3 was also significantly higher than that in the control group (*p* < 0.05) between WK4 to WK6. However, no differences were observed in the concentrations of IgA and IgM in the control group and group T1 between WK2 to WK8 (Table 3); no differences were observed in the concentrations of IgG between groups T1, T2, and control group at WK2 (Table 3). In addition, treatment and time had a significant effect on serum IgA, IgM, and IgG concentrations, but no differences were identified with respect to the interaction of treatment and time.

**TABLE 3:** The serum immunoglobulin concentrations in Holstein dairy calves fed with different doses of multispecies probiotics.

**Items**	**Treatment (Trt)^1^**				**SEM**	***p*-Value**					
	**C**	**T1**	**T2**	**T3**		**Trt**	**Time**	**Trt × Time**	**C × T1**	**C × T2**	**C × T3**
IgA, μg/ml											
WK2	2,608.6^c^	2,939.47^bc^	3,351.6^ab^	3,699.25^a^	150.30	<0.01	0.05	0.97	0.27	0.02	<0.01
WK4	2,822.91^b^	3,041.56^b^	3,445.02^a^	3,774.15^a^	120.81				0.21	<0.01	<0.01
WK6	3,030.28^c^	3,258.77^bc^	3,572.28^ab^	3,975.74^a^	120.78				0.254	0.02	<0.01
WK8	2,984.88^b^	3,301.57^ab^	3,399.9^ab^	3,758.25^a^	117.73				0.28	0.17	0.02
IgM, μg/ml											
WK2	1,464.23^c^	1,719.11^bc^	2,118.18^a^	1,940.73^ab^	83.99	<0.01	<0.01	0.14	0.09	<0.01	<0.01
WK4	1,775.56^b^	2,099.36^a^	2,126.5^a^	2,287.51^a^	69.91				0.05	0.03	<0.01
WK6	1,789.02^b^	2,153.23^ab^	1,988.26^b^	2,529.92^a^	96.30				0.061	0.27	<0.01
WK8	1,849.23^c^	2,058.37^b^	2,420^a^	2,287.9^a^	70.53				0.03	<0.01	<0.01
IgG, mg/ml											
WK2	8.72	10.89	12.93	11.48	0.87	<0.01	<0.01	0.21	0.40	0.12	0.29
WK4	9.08^b^	13.52^ab^	13.71^ab^	16.92^a^	1.07				0.08	0.07	<0.01
WK6	12.16^b^	12.96^b^	13.81^b^	18.51^a^	0.92				0.67	0.39	<0.01
WK8	14.3^b^	13.93^b^	15.05^b^	17.38^a^	0.49				0.70	0.44	0.01

*^*a*–*c*^The values in the same row with different superscripts are significantly different (*p* < 0.05), while values with the same or no superscripts mean no significant difference (*p* > 0.05).^1^Treatments: C, no supplementation (control); T1 = 0.5 g/calf/day; T2 = 1 g/calf/day; T3 = 2 g/calf/day.IgA, immunoglobulin A; IgM, immunoglobulin M; IgG, immunoglobulin G; SEM, standard error of the mean.*

### Fecal Microbial Diversity

In this study, 16S rRNA and ITS genes were amplified, and their sequences were analyzed to study the effect of probiotics on fecal microbiota of pre-weaning calves during the four time points (WK 2, 4, 6, and 8). A total of 2,907,932 high-quality sequences were obtained from the rectal samples, with an average of 37,862 sequences per sample (26,220–43,090 sequences).

The indicators of α diversity of bacteria showed that group T3 had a higher number of observed species and Shannon estimator than the control, T1, or T2 groups on WK2 (*p* ≤ 0.05, Table 4). The indicators of α diversity of fungi showed that group T3 had a higher number of ACE estimator than the control, T1, and T2 groups at WK2 (*p* ≤ 0.05, Table 5). In addition, time, and the interaction of treatment and time, had a significant effect on the α diversity of bacteria, whereas time had a significant effect on the α diversity of fungi. In addition, time, and the interaction of treatment and time, had a significant effect on α diversity of bacteria and fungi, but no differences were identified with respect to the treatment and time having a significant effect on the α diversity of fungi.

**TABLE 4:** The abundance and diversity of microbial microbiota in Holstein dairy calves fed with different doses of multispecies probiotics.

**Items**	**Treatment (Trt)^1^**				**SEM^2^**	***p*-Value**					
	**C**	**T1**	**T2**	**T3**		**Trt**	**Time**	**Trt × Time**	**C × T1**	**C × T2**	**C × T3**
Bacteria											
Observed species											
WK2	105.00	140.25	124.75	153.50	8.6	<0.01	<0.01	<0.01	0.57	0.23	0.05
WK4	309.75	356.75	347.00	324.75	11.24				0.17	0.27	0.65
WK6	362.25^ab^	378^ab^	322.5^b^	404.25^a^	12.65				0.63	0.236	0.21
WK8	369.25^a^	414^a^	413.5^a^	170^b^	28.19				0.21	0.22	<0.01
Chao1 index											
WK2	194.80	203.37	201.63	207.16	10.72	<0.01	<0.01	<0.01	0.80	0.84	0.72
WK4	365.86	402.29	399.33	361.64	11.75				0.30	0.34	0.90
WK6	412.28^ab^	422.08^ab^	372.88^b^	457.99^a^	12.76				0.76	0.24	0.17
WK8	408.69^a^	454.05^a^	458.99^a^	203.68^b^	29.4				0.24	0.20	<0.01
Shannon index											
WK2	2.28^ab^	1.77^b^	2.17^b^	2.79^a^	0.13	<0.38	<0.01	<0.01	<0.01	0.05	0.10
WK4	3.83	3.90	3.98	3.87	0.39				0.80	0.63	0.90
WK6	3.99^ab^	4.30^a^	3.68^b^	4.19^ab^	0.1				0.26	0.24	0.46
WK8	3.94^ab^	4.26^a^	4.29^a^	3.09^b^	0.18				0.46	0.42	0.06
Simpson index											
WK2	0.19^ab^	0.30^a^	0.22^ab^	0.13^b^	0.02	<0.01	0.09	<0.01	0.01	0.13	0.33
WK4	0.05	0.07	0.04	0.06	0.01				0.47	0.85	0.77
WK6	0.06	0.03	0.07	0.04	0.008				0.19	0.61	0.36
WK8	0.06	0.04	0.04	0.15	0.02				0.71	0.70	0.20
ACE index											
WK2	186.58	217.03	200.34	245.47	12.42	0.01	<0.01	<0.01	0.40	0.70	0.12
WK4	362.02	400.26	389.64	359.31	11.04				0.25	0.40	0.93
WK6	405.99^ab^	414.34^ab^	367.31^b^	452.11^a^	12.24				0.78	0.22	0.14
WK8	411.65^a^	452.07^a^	455.97^a^	195.64^b^	29.93				0.28	0.24	<0.01
Fungi											
Observed species											
WK2	19.50^b^	66.75^a^	43.00^ab^	45.00^ab^	6.60	0.39	<0.01	<0.01	0.80	0.77	
WK4	41.25	80.50	80.75	59.25	6.98				0.04	0.04	
WK6	132.50^a^	61.50^b^	62.50^b^	99.75^ab^	10.85				0.13	0.14	
WK8	134.25	131.50	125.50	51.00	15.46				0.95	0.83	
Chao1 index											
WK2	20.50^b^	74.58^a^	48.10^ab^	51.18^ab^	7.46	0.43	<0.01	0.03	0.01	0.14	
WK4	45.64	95.51	89.68	72.67	8.04				0.06	0.05	
WK6	152.22^a^	69.41^b^	93.25^ab^	109.06^ab^	13.20				0.03	0.10	
WK8	149.94	149.61	141.48	62.92	17.67				0.99	0.86	
Shannon index											
WK2	1.03^b^	1.85^ab^	2.01^a^	1.79^ab^	0.16	0.36	0.01	0.17	0.07	0.03	
WK4	2.01^b^	2.06^ab^	2.53^a^	2.44^ab^	0.09				0.79	0.03	
WK6	2.47	2.30	2.16	1.71	0.18				0.75	0.56	
WK8	2.30	2.31	2.28	1.97	0.08				0.97	0.94	
Simpson index											
WK2	0.52	0.33	0.26	0.30	0.05	0.67	0.02	0.15	0.16	0.06	
WK4	0.26	0.23	0.16	0.16	0.02				0.52	0.05	
WK6	0.20	0.22	0.31	0.40	0.04				0.88	0.35	
WK8	0.22	0.25	0.24	0.27	0.02				0.60	0.73	
ACE index											
WK2	20.83^b^	83.20^a^	48.07^ab^	56.12^ab^	8.03	0.31	<0.01	0.03	<0.01	0.15	
WK4	45.64^b^	95.51^a^	89.68^ab^	72.67^ab^	8.04				0.028	0.047	
WK6	146.15	84.74	110.37	113.63	10.92				0.06	0.25	
WK8	149.29	152.97	142.02	63.25	17.24				0.94	0.87	

*^*a,b*^The values in the same row with different superscripts are significantly different (*p* < 0.05), while values with the same or no superscripts mean no significant difference (*p* > 0.05).^1^Treatments: C, no supplementation (control); T1 = 0.5 g/calf/day; T2 = 1 g/calf/day; T3 = 2 g/calf/day.SEM, standard error of mean.*

**TABLE 5:** Analysis of similarity (ANOSIM) of multispecies probiotics on fecal bacteria and fungi of Holstein dairy calves.

**Variable^1^**	**Bray–Curtis ANOSIM**	***p*-Value^2^**	**Binary Jaccard ANOSIM**	***p*-Value^2^**
**Bacteria**				
WK2	0.732	0.001	0.532	0.001
WK4	0.077	NS	0.027	NS
WK6	0.526	0.001	0.358	0.001
WK8	0.373	0.001	0.381	0.002
Fungi				
WK2	0.265	0.009	0.139	0.084
WK4	0.281	0.013	0.279	0.002
WK6	−0.030	NS	0.171	0.032
WK8	0.308	0.007	0.212	0.087

*^1^Directly fed compound probiotics for pre-weaning calves of different ages at WK2, WK4, WK6, and WK8.^2^NS, not significant (*p* > 0.05). 1 > *r* > 0, indicates a significant difference between groups; −1 < *r* < 0 indicates that the difference between groups is not significant.*

**FIGURE 1:**
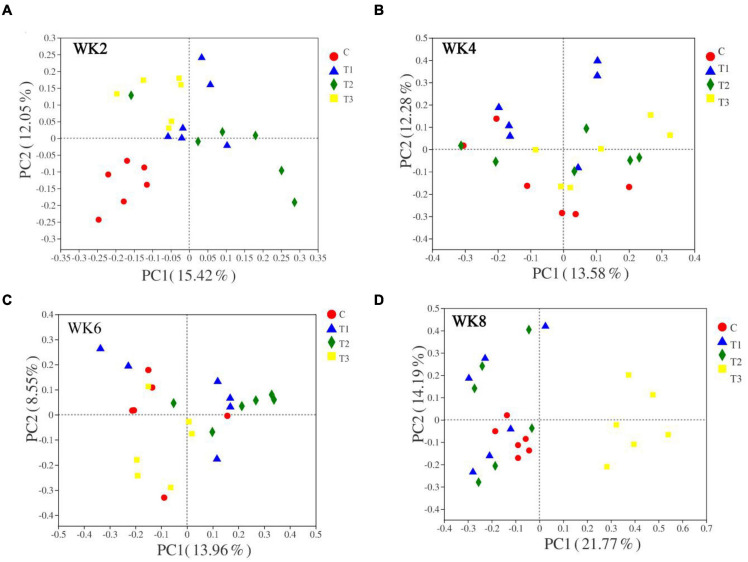
Based on Bray–Curtis distance principal coordinates analysis (PCoA) (Panels **A–D** indicate WK2, WK4, WK6, and WK8 bacteria PCoA pictures).

**FIGURE 2:**
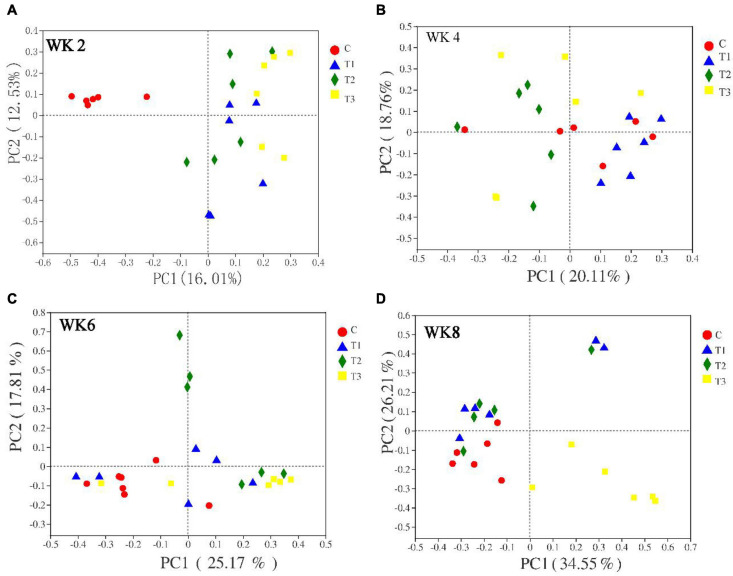
Based on Bray–Curtis distance principal coordinates analysis (PCoA) (Panels **A–D** indicate WK2, WK4, WK6, and WK8 fungi PCoA pictures).

A plot of the principal coordinate analysis scores showed great similarity between group 4 of fecal microbiota at WK4, WK6, and WK8 (Figures 1, 2). The diversity of the microbiota in group T1 was similar to that in the control group at WK4, WK6, and WK8, whereas that of group T3 demonstrated a marked shift along principal component 1 when compared with the control group (Figures 1, 2).

The bacterial communities in the feces samples of calves at different weeks showed that the bacterial communities in WK2 (Bray–Curtis analysis of similarity or ANOSIM = 0.732), WK6 (Bray–Curtis ANOSIM = 0.526), and WK8 (Bray–Curtis ANOSIM = 0.373) were clustered. The clustering of fungi communities in WK2 (Bray–Curtis ANOSIM = 0.265), WK4 (Bray–Curtis ANOSIM = 0.281), and WK8 (Bray–Curtis ANOSIM = 0.308) indicates that the inter-group difference is more significant than the intra-group difference. There were no significant differences between the bacteria in WK4 (Bray–Curtis ANOSIM = 0.077) and fungi in WK6 (Bray–Curtis ANOSIM = −0.030). Principal coordinates analysis (PCoA) and ANOSIM analyses revealed that adding different doses of MSP during the feeding process of pre-weaning calves exert significant differences in the microbial structure of the fecal microbiota (Table 5).

### Relative Abundance of Bacterial and Fungal Taxa

**FIGURE 3:**
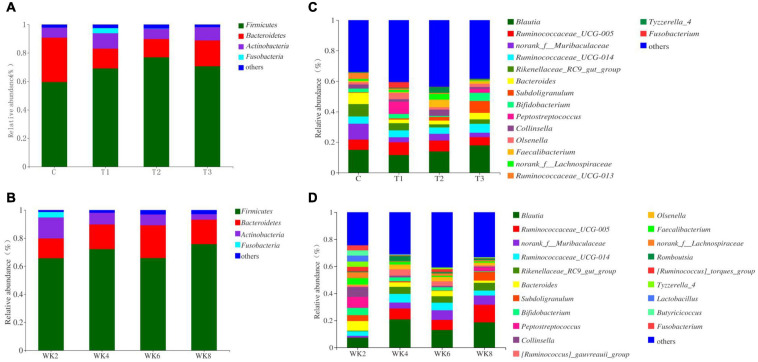
Composition of the fecal microbial community. Panels **(A,B)** show the composition of the microbial community in the four groups (C, T1, T2, and T3) with respect to the phyla and genera of bacterial. Panels **(C,D)** show the compositions at four time points during the study (WK 2, 4, 6, and 8).

A comparison of the effects of groups T1, T2, and T3 supplementation on the fecal microbial composition of pre-weaning calves on different weeks was carried out using a taxon-dependent analysis. *Firmicutes* and *Bacteroidetes* were the dominant bacteria phyla, followed by *Actinobacteria*, *Proteobacteria*, and *Tenericutes* (Figures 3A,B). The relative abundance of *Firmicutes* was identified for calves of T3 group compared with the control group at WK8 (*p* < 0.01; Supplementary Table 1). However, as the calves grew older and treatments continued, no significant changes were observed in the relative abundance across the phyla except for *Bacteroidetes* and *Fusobacteria* (Figures 3A,B and Supplementary Table 2). *Blautia* and *Ruminococcaceae_UCG005* were the predominant bacteria genera, followed by *Ruminococcaceae_UCG-014*, *Rikenellaceae_RC9_gut_group*, *Bacteroides*, *Subdoligranulum*, *Bifidobacterium*, *Peptostreptococcus*, *Collinsella*, *Lactobacillus*, *Butyricicoccus*, and *Dorea* (Figures 3C,D). The relative abundances of *Bifidobacterium* and *Ruminococcaceae_UCG-014* tended to be higher in group T3 than in the control, T1, and T2 groups at WK2 (*p* = 0.05, *p* = 0.03, respectively; Supplementary Table 2). In addition, time had no significant effect on the representation of genera except for *Blautia*, *Collinsella*, *Lactobacillus*, *Butyricicoccus*, and *Dorea* (*p* = 0.03, *p* < 0.01, *P* < 0.01, *P* = 0.01, *P* < 0.01, respectively). Treatment and treatment × time had no significant effect on the representation of genera except for the *Ruminococcaceae_UCG005*, *norank_f__Muribaculaceae*, *Rikenellaceae_RC9_gut_group*, *Peptostreptococcus*, or *[Ruminococcus]_gauvreauii_group* genera (*P* < 0.01, respectively).

**FIGURE 4:**
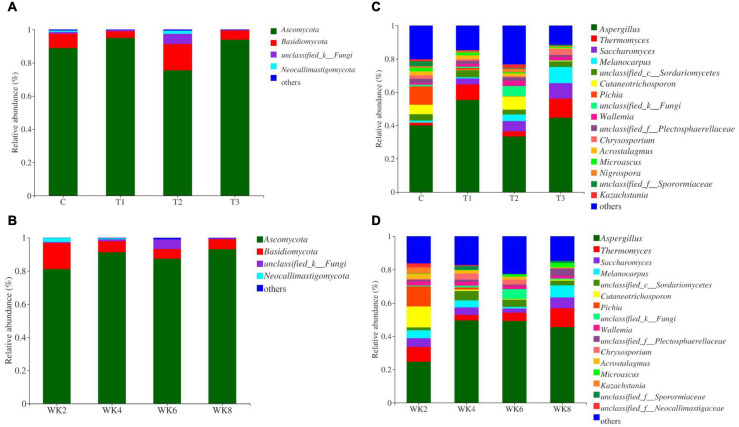
Composition of the fecal microbial community. Panels **(A,B)** show the composition of the microbial community in the four groups (C, T1, T2, and T3) with respect to the phyla and genera of fungi. Panels **(C,D)** show the compositions at four time points during the study (WK 2, 4, 6, and 8).

*Ascomycota* and *Basidiomycota* were the dominant fungal phyla in calf rectal microbial composition (Figures 4A,B), followed by *Neocallimastigomycota* and unclassified fungi. However, as the calves grew older, time and treatment had no significant effect on the relative abundance of fungal phyla. *Aspergillus* and *Thermomyces* were the predominant fungal genera, followed by *Saccharomyces, Melanocarpus*, *Cutaneotrichosporon*, *Pichia*, *Wallemia*, *Chrysosporium*, *Acrostalagmus*, *Microascus*, *Nigrospora*, and *Kazachstania* (Figures 4C,D). The relative abundances of *Aspergillus*, *Saccharomyces*, *Melanocarpus*, and *Chrysosporium* tended to be higher in the T3 group than that in the control, T1, and T2 group at WK2 (*p* = 0.03; *p* = 0.01; *p* = 0.05; *p* = 0.03, respectively; Supplementary Table 2). The relative abundances of *Pichia* tended to be lower in the group T3 than that in the control group at WK2 (*p* < 0.05, Supplementary Table 2). In addition, time had no significant effect on the representation of each genera except for the relative abundance of *Microascus* and *unclassified_k__Fungi* (*p* < 0.01; *p* = 0.02; respectively). Time and trt-time had no significant effect on the representation across genera except for *Cutaneotrichosporon* (*p* = 0.02, *p* = 0.03, respectively).

### Relationships Between the Size of Microbial Populations and Immunoglobulin Indices

We next analyzed the relationship between the size of each fecal microbial population at WK2, and both growth performance and immunoglobulin indices using Pearson’s rank correlation coefficients. At the genus level, the relative abundance of *Bifidobacterium* positively correlated with ADG concentrations (*p* < 0.05; Table 6), while the relative abundance of *Melanocarpus* positively correlated with total feed intake and starter intake (*p* < 0.05), and the relative abundance of *Saccharomyces* positively correlated with IgA and IgG (*p* < 0.05).

**TABLE 6:** Pearson correlation coefficients between the populations of the principal genera at WK 2 and immunoglobulin indices.

**Genus**	**ADG**	**Total feed intake**	**Starter intake**	**Feed efficiency**	**Serum concentration**		
					**IgA**	**IgG**	**IgM**
Bacteria							
*Blautia*	0.18	−0.71*	−0.71*	0.21	–0.35	−0.57*	–0.39
*Bacteroides*	0.20	–0.37	–0.37	0.36	−0.72*	–0.28	–0.37
*Ruminococcaceae_UCG-014*	0.43	0.28	0.28	0.14	0.37	0.19	0.26
*Peptostreptococcus*	0.07	0.37	0.37	–0.29	–0.15	0.05	–0.12
*Bifidobacterium*	0.34*	0.05	0.05	0.32	0.38	0.00	0.09
*Collinsella*	−0.54*	–0.25	–0.25	–0.26	–0.18	–0.02	0.09
*Faecalibacterium*	–0.07	0.00	0.00	0.21	0.33	0.00	0.24
*Subdoligranulum*	–0.04	0.02	0.02	0.13	0.37	0.22	0.40
*Lactobacillus*	–0.15	0.16	0.16	0.12	0.01	0.25	0.10
*Norank_f__Lachnospiraceae*	–0.28	–0.09	–0.09	–0.58	–0.23	0.28	0.30
*Tyzzerella_4*	–0.41	–0.02	–0.02	–0.03	0.24	0.39	0.40
*Fusobacterium*	0.01	0.06	0.06	–0.14	–0.21	–0.18	–0.21
*Butyricicoccus*	–0.34	–0.38	–0.38	–0.20	–0.29	–0.39	–0.42
*[Ruminococcus]_torques_group*	–0.49	–0.05	–0.05	–0.19	–0.21	–0.07	–0.02
*Dorea*	–0.36	–0.05	–0.05	0.18	0.33	0.46	0.44
*Corynebacterium_1*	0.18	0.05	0.05	–0.04	0.12	0.02	–0.10
*Peptoclostridium*	–0.08	–0.04	–0.04	–0.04	0.18	0.12	0.21
*Prevotella_7*	0.24	0.17	0.17	0.22	0.38	0.14	0.25
*Norank_f__Muribaculaceae*	0.27	0.19	0.19	0.36	0.13	–0.02	–0.33
*Peptostreptococcus*	0.07	0.37	0.37	–0.29	–0.15	0.05	–0.12
Fungi							
*Aspergillus*	0.23	0.38	0.38	0.04	0.28	0.00	0.22
*Cutaneotrichosporon*	–0.29	−0.60**	−0.60**	–0.22	–0.30	–0.24	0.11
*Pichia*	0.15	–0.21	–0.21	–0.65	−0.55*	–0.40	–0.45
*Thermomyces*	0.09	0.50	0.50	0.02	0.23	0.23	0.26
*Saccharomyces*	–0.15	0.23	0.23	0.05	0.52*	0.56*	0.73
*Melanocarpus*	0.30	0.51*	0.51*	0.24	0.45	0.24	0.26
*Kazachstania*	–0.27	0.21	0.21	–0.14	0.01	0.47	0.09
*Acrostalagmus*	0.23	0.05	0.05	–0.06	–0.24	–0.03	–0.12
*Unclassified_f__Neocallimastigaceae*	–0.18	0.18	0.18	0.09	0.05	0.39	0.02
*Wallemia*	–0.04	0.11	0.11	0.06	0.40	0.11	0.45
*Unclassified_o__Chaetothyriales*	–0.07	–0.05	–0.05	0.04	−0.35*	–0.01	–0.53
*Unclassified_c__Sordariomycetes*	0.05	0.27	0.27	0.12	0.51	0.42	0.57
*Unclassified_p__Ascomycota*	–0.40	0.14	0.14	–0.29	–0.15	0.34	–0.08
*Candida*	0.42	–0.05	–0.05	–0.21	–0.43	–0.29	–0.42
*Ramularia*	–0.37	0.25	0.25	0.28	–0.08	0.36	–0.31
*Unclassified_f__Plectosphaerellaceae*	–08	0.04	0.04	0.33	0.46	0.30	0.59
*Veronaea*	–0.09	–0.14	–0.14	0.17	0.25	0.08	0.25
*Paraphaeosphaeria*	–0.30	0.12	0.12	–0.23	–0.27	0.21	–0.12
*Acremonium*	–0.20	0.23	0.23	0.25	0.24	0.28	–0.15
*Unclassified_k__Fungi*	–0.14	0.15	0.15	–0.05	–0.24	–0.08	0.08
*Nigrospora*	–0.14	0.21	0.21	–0.12	0.22	0.38	0.41
*Unclassified_o__Pleosporales*	0.18	0.14	0.14	0.11	0.28	0.16	0.22
*Golovinomyces*	–0.26	–0.25	–0.25	–0.42	–0.14	0.31	0.36
*Chrysosporium*	0.39	0.41	0.41	0.26	0.28	0.00	0.22

*p < 0.05, **p < 0.01.

The relative abundance of *Blautia* correlated negatively with total feed intake, starter intake, and IgG (*p* < 0.05). The relative abundance of *Collinsella* correlated negatively with ADG concentrations (*p* < 0.05). The relative abundance of *Bacteroides* also showed a negative correlation with IgA (*p* < 0.05). A negative correlation was also found between the relative abundance of *Cutaneotrichosporon* and that of feed intake and starter intake concentrations (*p* < 0.01).

## Discussion

The gut microbial colonization of ruminants gradually colonizes from the fetal period to after birth (Klein-Jöbstl et al., 2019; Bi et al., 2021). Early gut microbiota plays a vital role in the long-term health of the host (Malmuthuge and Guan, 2017). The intestinal microbiota of newborn calves changes during the early postnatal period (Malmuthuge et al., 2015; Takino et al., 2017; Song et al., 2018; Klein-Jöbstl et al., 2019). Therefore, the probiotic supplementation provides opportunities to improve early-life gut health and to minimize calves’ susceptibility to enteric infections during the pre-weaning period (Van den Abbeele et al., 2011; Malmuthuge and Guan, 2017; Markowiak and Śliżewska, 2018).

The present study showed that supplementation with MSP in group T3, but not groups T1 and T2, significantly increased the ADG and total feed intake of newborn calves in the first 8 weeks after birth. *Lactobacillus acidophilus* (Bayatkouhsar et al., 2013; Foditsch et al., 2015; Sharma et al., 2018), *Bacillus subtilis* (Sun et al., 2010; Zhang et al., 2017), and *Saccharomyces cerevisiae* (Villot et al., 2019) had growth-promoting effects. Timmerman et al. (2005) also found that MSP-treated veal calves had growth-promoting effects over placebo-treated veal calves from day 1 to 56, but the results were not statistically significant. In the present study, calves gained 875 and 720 g/day of growth in the T1 and T3 group, respectively. This result is consistent with the findings of Renaud et al. (2019), who showed that calves gained 630 g/day of growth when receiving a 4-g bolus of the MSP. Notably, the total feed intake in the T3 group tended to be higher than in the T1, T2, and control groups, which might be due to the higher bioavailability of MSP that can produce organic acids and many kinds of metabolites in the process of animal metabolism enzymes and some important nutrients (Pandey et al., 2015). Differences in results between the different tests may be related to the type, quantity, proportion, and method of probiotic delivery, as well as the different management levels between the cattle farms.

In the present study, the incidence of diarrhea in control calves fluctuated between 1.14% and 25.11% during the first 4 weeks of life. However, supplementation with MSP was helpful to reduce the incidence of diarrhea in neonatal dairy calves during day 7–21 after birth (Ma et al., 2020), which is consistent with previous studies (Wehnes et al., 2009; Novak et al., 2012; Renaud et al., 2019). Prevention and control of outbreaks before occurring are more cost effective (Knights et al., 2011), and current studies have found that early intervention of probiotics has a better preventive effect (Hempel et al., 2012; Guarino et al., 2015; Hua et al., 2016). Malmuthuge and Griebel (2018) and other researchers have described the potential strategies for controlling early microbiota and to improve the health of newborn calves during the period when they are most susceptible to intestinal diseases. We found that compared with the control group, groups T1, T2, and T3 significantly reduced the diarrheal rates of calves that were 4 weeks old or less, but had no significant effect on those that were between WK6 and WK8 old. However, there is limited information on specific changes in fecal microbiota resulting from the direct feeding of multispecies probiotics to neonatal calves.

One of the recent studies have proposed that supplementation with *Saccharomyces cerevisiae* increased the immune responsiveness of calves by increasing IgA concentration (Villot et al., 2020). Furthermore, Sun et al. (2020) showed that *Bacillus subtilis natto* increased general performance by improving the ADG and feed efficiency, and advanced the weaning age of the calves. While there is no difference in serum IgA and IgM, serum IgG was higher in the *Bacillus subtilis natto*-supplemented calves than in the control calves. Consistent with these findings, we found that MSP supplementation in the T3 group increased serum IgA, IgM, and IgG concentrations above those of the control by 1.1, 0.48, and 2.76 mg/ml, respectively, compared with groups T1 and T2 supplementation, indicating that group T3 is superior to groups T1 and T2 with respect to the immune function of dairy calves.

We observed no significant difference in the Shannon index between WK2 and WK8 after adding multispecies probiotics. This finding is similar to the change in fecal microbiota in the first 8 weeks of calves reported earlier (Knights et al., 2011; Cho and Yoon, 2014). However, adding probiotics to the calves’ diet before weaning can change the bacterial diversity and composition of the gastrointestinal tract, but has little impact on the diversity and a greater impact on the composition of the microbial community (Villot et al., 2019). After adding MSP, we compared the outcomes with the control group. In WK2, the MSP supplementation increased the relative abundance of *Firmicutes* and significantly reduced the relative abundance of *Bacteroidetes*. *Firmicutes*, *Bacteroidetes*, and *Actinobacteria* are the dominant microbial taxa in the hindgut of pre-weaning calves and humans (Song et al., 2018; Kassaian et al., 2020). *Firmicutes* is often the dominant phylum in most animal species (Guarino et al., 2015). In diarrheic intestines of children, *Bacteroides* remain the dominant genera. In this study, probiotics significantly reduced the relative abundance of *Bacteroides* while increasing the relative abundance of *Ruminococcaceae_UCG-005* (Saraf et al., 2017).

The representative genera from *Bifidobacterium*, *Lactobacillus*, *Subdoligranulum*, *Blautia*, and *Bacteroides* were closely related to healthy calves (Jang et al., 2019; Schwaiger et al., 2020), which is consistent with the findings of our study. The presence of *Bifidobacterium* family D7 at birth is similar to the fecal microbiota of vaginal delivery in infants (Kassaian et al., 2020) and the intestinal tract of early infants (Arrieta et al., 2014). In this study, the abundance of *Bifidobacterium* was higher at WK2 than in the older calves (WK4, WK6, and WK8). *Bifidobacteria* plays an important role in immune stimulation in host invasion (Hidalgo-Cantabrana et al., 2014). Additionally, the gene expression and microRNA expression in the small intestine of the same calf were highly correlated to the number of *Bifidobacteria* (Liang et al., 2014). Therefore, it is important to know how the diversity of *Bifidobacterium* could be impacted by age and how this influences host functions. The differences in the composition of intestinal microbiota may not be the cause of diarrhea as some changes in bacterial abundance may guide our interpretation of diarrhea. This study provides a theoretical basis for the establishment of a control system for calf diarrhea (Arrieta et al., 2014).

The lower relative abundance of *Blautia* in diarrhea from 14-day-old calves when compared with healthy calves suggests that this genus may be associated with diarrhea (Ma et al., 2020). A high prevalence of *Blautia* has also been reported in the colon and feces of healthy neonatal swine (Saraf et al., 2017) and human infants (Jost et al., 2014; Sagheddu et al., 2017). However, fecal samples of dogs with diarrhea showed a general reduction of *Blautia. Blautia* utilizes polysaccharides that cannot be used by other intestinal microorganisms to degrade and produce butyrate, which is one of the main short-chain fatty acids that maintains intestinal health and the intestinal epithelial barrier by regulating the immune system (Guarino et al., 2015). *Collinsella* can metabolize carbohydrates of plant or animal origin, and together with *Bifidobacterium*, can modify the bile acids of the host, modulating the virulence and pathogenicity of enteric pathogens (Bag et al., 2017). The relative abundances of these bacteria in this study were observed to be higher in calves supplemented with MSP at WK2. Yet, the reduction in the abundance of this genus was reported in fecal samples of dogs with diarrhea.

The abundance of *Cutaneotrichosporon* observed in the control group was significantly lower than that observed in group T3. *Cutaneotrichosporon* have been described as a lipolytic yeast species from food and food-related environments (Péter et al., 2019). *Cutaneotrichosporon debeurmannianum* is a rarely isolated yeast from human blood and urine samples, with clinical samples coming from patients that were diagnosed with septicemia and urinary tract infections. The pathogenic potential and epidemiological relevance of this yeast remains to be seen (do Espírito Santo et al., 2020). In this study, the reduced abundance of *Cutaneotrichosporon* in group T3 did lead to an increase in total feed and starter intake than that of the control group. This suggests that *Cutaneotrichosporon* in calves influences the total feed intake and starter intake, but weirdly affects other aspects as they too have also been found to cause the occurrence of diarrhea.

The abundance of *Saccharomyces* observed in group T3 was significantly higher than that observed in the control group. *Saccharomyces* is rich in digestible proteins, vitamins (vitamin B6, thiamin, biotin, riboflavin, nicotinic acid, and pantothenic acid), magnesium, and zinc (Massé and Weiser, 1994). *Saccharomyces cerevisiae* flows along the gastrointestinal tract without adhering to its walls. The strains that do not have the ability to adhere to the intestinal epithelium, which are effective as bioregulators and their action are based on the ability of colonization through several mechanisms (Rodrigues et al., 2000; Baptista, 2002). In their study, the use of live yeast cells is to act as detoxification agents against mycotoxins, and other bacterial toxins and their receptors in the mucous membrane, and *Vibrio cholerae* toxin. Severe damage to organs has been eliminated due to diets that may contain these toxins in the presence of *S. cerevisiae* for their ability to reduce animal stress, providing vitamins, enzymes, and proteins (Baptista et al., 2005). *Saccharomyces cerevisiae* in calf diets augment immunological effect by increasing IgM and IgA activity against pathogens, enhancing intestinal development and function, adsorb mycotoxins, modulate gut microbiota, and reduce post-weaning diarrhea (Qamar et al., 2001; Sun et al., 2019; Elghandour et al., 2020). We found that the increased abundance of *Saccharomyces* in group T3 improved the concentrations of IgA and IgG than the control group. This result further supports the hypothesis that adding MSP may affect overall IgA and IgG serum concentrations by influencing the fecal microbial composition in calves.

## Conclusion

We demonstrated that supplementation with MSP in the T1, T2, and T3 groups had more advantages than the control group in terms of promoting growth performance and reducing the incidence of diarrhea in pre-weaning dairy calves. However, no significant differences were identified between the MSP and control groups with respect to these end points. The dose of MSP supplementation in group T3 had more advantages in reducing the incidence of diarrhea from WK2 to WK4 in newborn calves than groups T1, T2, and the control. Supplementation with MSP increased serum IgA and IgM concentrations in group T3 to levels that were significantly higher than those in the control group between WK2 and WK8. This implies that group T3 has relatively higher bioavailability than groups T1 and T2. Therefore, it indicated that the lower incidence of diarrhea in calves receiving MSP supplement is the result of an effect on fecal microbial composition and diversity. In view of their differing effects, we recommend adding 2 g/day of MSP supplementation in diets of dairy calves during their first 4 weeks of life before weaning. Our findings provide a basis for the rational use of MSP supplementation in calf production and may help to reduce the use of antibacterial agents.

## Data Availability Statement

The datasets presented in this study can be found in online repositories. The names of the repository/repositories and accession number(s) can be found in the article/Supplementary Material.

## Ethics Statement

The animal study was reviewed and approved by the Ethics Committee of the College of Animal Science and Technology, Shihezi University (No. A2019-155-01).

## Author Contributions

YW and WZ carried out the experimental design of this study. YW, RL, and HC contributed to the experimental implementation. YW and XL contributed to sampling of this study. YW and CN contributed to the data analysis. YW, LW, JN, CC, and YX contributed to the article writing. All authors contributed to the article and approved the submitted version.

## Conflict of Interest

RL and HC were employed by the company Xinjiang Tianshan Junken Animal Husbandry Co., Ltd. The remaining authors declare that the research was conducted in the absence of any commercial or financial relationships that could be construed as a potential conflict of interest.
