# Supplementary material for: Effect of a Multispecies Probiotic Mixture on the Growth and Incidence of Diarrhea, Immune Function, and Fecal Microbiota of Pre-weaning Dairy Calves
Source: Front Microbiol. 2021 Jul 14;12:681014. doi: 10.3389/fmicb.2021.681014 (PMC8318002; doi:10.3389/fmicb.2021.681014)
Supplement: Supplementary file 1 [file Data_Sheet_1.pdf]

Effect of a Multispecies Probiotic Mixture on the growth and incidence of diarrhea, immune function, and fecal microbiota of pre-weaning dairy calves

Yanyan Wu[1], Lili Wang[2], Ruiqing Luo[3], Hongli Chen[3], Cunxi Nie[1], Junli Niu[1], Cheng Chen [1], Yongping Xu[2], Xiaoyu Li[2], Wenju Zhang*[1]

[1]College of Animal Science & Technology, Shihezi University, Shihezi, 832003, China;

[2]School of Bioengineering, Dalian University of Technology, Dalian, 116024, China

[3]Xinjiang Tianshan Junken Animal Husbandry Co., Ltd. Shihezi , 832003, China

*[1]Corresponding author: Wenjun Zhang, College of Animal Science & Technology, Shihezi University, North 4th Road, Shihezi, 832003, China

(E-mail: zhangwj1022@sina.com)

Supplementary material

Supplementary Table 1. Taxonomic analysis of the principal fecal phlya in Holstein dairy calves, classified according to multispecies probiotics and time point

Phylum (%)	Treatment (Trt)[1]				SEM[2]	*P*-value					
	C	T1	T2	T3		Trt	Time	Trt×Time	C×T1	C×T2	C×T3
Bacteria											
Firmicutes											
WK2	73.29	69.70	61.45	71.45	4.99	0.45	0.24	0.08	0.82	0.46	0.91
WK4	79.24	71.71	61.00	76.87	3.23				0.39	0.05	0.78
WK6	63.24	74.36	63.89	59.97	3.90				0.35	0.96	0.78
WK8	49.73[b]	78.68[a]	76.31[a]	90.52[a]	4.40				< 0.01	< 0.01	< 0.01
Bacteroidetes											
WK2	17.23	17.06	8.90	17.86	4.09	0.36	0.31	<0.01	0.99	0.52	0.96
WK4	10.51[b]	13.21[ab]	28.24[a]	18.11[ab]	2.50				0.65	0.01	0.22
WK6	19.37	18.64	27.52	29.15	3.54				0.95	0.45	0.37
WK8	45.42a	15.51b	17.16b	0.21c	4.59				< 0.01	< 0.01	< 0.01
Actinobacteria											
WK2	13.43	11.27	29.09	5.87	0.35	0.64	0.06	0.29	0.87	0.25	0.57
WK4	8.26	12.61	8.86	3.56	2.29				0.53	0.93	0.50

WK6	14.27	5.00	4.91	5.85	2.35				0.19	0.19	0.24
WK8	1.01	2.59	2.29	8.99	1.47				0.69	0.75	0.06
Proteobacteria											
WK2	0.48	1.69	0.19	2.05	0.54	0.20	0.25	0.26	0.46	0.86	0.35
WK4	0.34	0.04	0.15	3.78	0.89				0.90	0.95	0.21
WK6	0.09	0.15	0.18	0.08	0.01				0.23	0.09	0.84
WK8	0.18	0.10	0.16	0.10	0.02				0.30	0.80	0.32
Fusobacteria											
WK2	0.05	0.02	0.61	0.07	0.15	< 0.01	< 0.01	< 0.01	0.96	0.18	0.88
WK4	0.00	0.00	0.00	0.00	0.00				1	1	1
WK6	0.00	0.00	0.00	0.00	0.00				1	1	1
WK8	0.00	0.00	0.00	0.00	0.00				1	1	1
Tenericutes											
WK2						0.54	0.60	0.37			
WK4	0.38	1.29	0.91	0.73	0.19				0.137	0.372	0.548
WK6	1.66	0.83	0.82	1.14	0.28				0.346	0.336	0.55
WK8	1.46	1.37	3.18	0.23	0.61				0.959	0.312	0.495
Patescibacteria											
WK2											
WK4	1.14	0.25	0.20	0.12	0.19	0.47	0.65	0.08			
WK6	0.37	0.02	0.09	0.43	0.12				0.39	0.484	0.879
WK8	0.02	0.62	0.33	0.00	0.12				0.094	0.361	0.95
Fungi											
Ascomycota											

WK2	61.67	97.06	84.77	57.81	8.58	0.69	0.06	0.19	0.16	0.35	0.87
WK4	92.35	91.52	94.07	93.14	1.33				0.84	0.68	0.86
WK6	96.54	70.43	91.35	91.25	5.25				0.10	0.73	0.72
WK8	96.09	91.30	95.48	88.12	1.24				0.12	0.84	0.02
Basidiomycota											
WK2	4.89	2.88	13.62	41.54	6.90	0.07	0.10	0.14	0.91	0.63	0.06
WK4	5.33	5.05	5.16	6.72	0.86				0.92	0.95	0.63
WK6	3.00	5.42	8.33	6.59	1.32				0.54	0.19	0.37
WK8	3.56	8.25	4.06	9.61	1.11				0.12	0.86	0.05
unclassified_k__Fungi											
WK2	0.48	0.00	1.61	0.13	0.41	0.46	0.44	0.38	0.69	0.36	0.77
WK4	2.32	0.34	0.61	0.11	0.57				0.26	0.32	0.21
WK6	0.47	22.50	0.28	0.33	5.43				0.18	0.99	0.99
WK8	0.34	0.40	0.41	1.81	0.24				0.92	0.91	0.02
Neocallimastigomycota											
WK2	7.96	0.00	0.00	0.01	1.99	0.49	0.49	0.41	0.18	0.18	0.18
WK4	0.00	2.23	0.00	0.00	0.56				0.18	1.00	1.00
WK6	0.00	0.00	0.00	0.00	0.00				0.00	0.00	0.00
WK8	0.00	0.00	0.00	0.00	0.00				0.00	0.00	0.00

[a-b] The values in the same row with different superscripts are significantly different ($P < 0.05$), while values with the same or no superscripts mean no significant difference ($P > 0.05$).

[1]Treatments: C = no supplementation (control); T1 = 0.5 g/calf/day; T2 =1 g/calf/day; T3 = 2 g/calf/day.

[2]SEM = Standard error of mean.

Supplementary Table 2. Taxonomic analysis of the principal fecal genera in Holstein dairy calves, classified according to multispecies probiotics and time point

Genus (%)	Treatment (Trt)[1]				SEM[2]	*P*-value					
	C	T1	T2	T3		Trt	Time	Trt×Time	C×T1	C×T2	C×T3
Bacteria											
Blautia											
WK2	4.275	4.5633	10.4275	11.0475	3.06	0.38	0.03	0.84	0.977	0.513	0.472
WK4	17.1725	22.6225	21.37	22.2125	2.52				0.896	0.948	0.915
WK6	11.435	16.7625	7.7875	14.725	2.26				0.436	0.591	0.628
WK8	15.7375	27.765	6.87	20.7375	4.18				0.324	0.463	0.677
Collinsella											
WK2	11.535	4.9725	7.4925	4.975	1.93	0.53	<0.01	0.79	0.272	0.492	0.273
WK4	0	0.64	0.71	0	0.23				0.781	0.724	1
WK6	2.775	0	0	0.5175	0.69				0.19	0.19	0.28
WK8	0.0375	0.105	0	1.7175	0.42				0.957	0.976	0.193
Bacteroides											
WK2	7.6875	5.585	5.3875	9.6325	2.17	0.79	0.05	0.89	0.76	0.738	0.777
WK4	1.8275	2.6725	2.6775	5.4475	0.79				0.981	0.981	0.407
WK6	2.775	5.5925	2.75	4.095	0.85				0.285	0.992	0.61
WK8	0.7625	0	4.8275	1.03	0.74				0.677	0.042	0.883
Bifidobacterium											
WK2	0.1	0.4	0.51	20.39	4.64	0.65	0.2	0.47	0.982	0.141	0.05

WK4	1.2775	0	0	9.2175	2.3				0.997	0.634	0.997
WK6	0.485	0	8.7475	0	2.18				0.939	0.939	0.207
WK8	0.0725	0.2275	0	1.2525					0.788	0.75	0.9
Faecalibacterium											
WK2	3.79	0.8075	13.92	3.18	0.12	0.23	0.81	0.76	0.987	0.178	3.79
WK4	1.635	0.4775	3.2725	0.83					0.997	0.554	1.635
WK6	0	0	1.15	0.22					1	0.031	0
WK8	0	0	2.9575	0.73					1	0.183	0
Lactobacillus											
WK2	3.4225	2.5875	9.1125	2.48	1.7	0.51	<0.01	0.56	0.868	0.268	0.851
WK4	2.855	0	0	0	0.35				0.515	0.515	0.515
WK6	0	0	0	0							
WK8	0	0	0	0							
Subdoligranulum											
WK2	5.725	2.6075	8.79	0.225	2.48	0.67	0.15	0.95	0.681	0.686	0.471
WK4	0	1.1125	0	0.765	0.32				0.659	1	0.853
WK6	0	0	0	0	0				0.906	0.672	0.38
WK8	9.4	8.245	5.2375	0.6575	3.15						
Tyzzerella											
WK2	4.5425	7.145	0.28	4.25	1.92	0.59	0.06	0.86	0.659	0.474	0.96
WK4	0	1.0525	0	0	0.17				0.121	1	1
WK6	0.9175	0	0	2.915	0.74				0.679	0.679	0.374
WK8	0.3775	0.715	0.44	0	0.21				0.614	0.925	0.573
Butyricicoccus											

WK2	4.78	7.3975	1.76	1.735	1.83	0.62	0.01	0.85	0.632	0.609	0.578
WK4	0.00	0.00	0.00	0.00					1.00	1.00	1.00
WK6	0.00	0.00	0.00	0.00					1.00	1.00	1.00
WK8	0.0725	0.3275	0	0.1475	0.08				0.344	0.784	0.777
Ruminococcus-2											
WK2	0	3.6325	0.425	0.325	0.25	0.33	0.24	0.63	0.084	0.83	0.869
WK4	1.2175	3.7625	4.045	0	0.79				0.623	0.544	0.934
WK6	0	1.6225	0	0.6675	0.42				0.215	1	0.6
WK8	0	1.095	2.12	0.2125	0.54				0.499	0.202	0.895
Dorea											
WK2	2.15	3.565	3.1075	1.8725	1.1	0.96	<0.01	0.99	0.689	0.786	0.937
WK4	0	0	0	0					1.00	1.00	1.00
WK6	0	0	0	0					1.00	1.00	1.00
WK8	0.52	0.3575	0.34	0.215	0.16				0.764	0.74	0.575
Ruminococcaceae_UCG005											
WK2	0.00	0.00	0.00	0.00	0.00	0.27	<0.01	<0.01	0.00	0.00	0.00
WK4	7.54	7.12	7.89	9.48	1.10				0.91	0.92	0.58
WK6	10.43	5.40	2.34	12.02	1.73				0.28	0.10	0.73
WK8	9.07	21.01	19.91	0.00	2.38				0.00	0.00	0.01
norank_f__Muribaculaceae											
WK2	2.97	0.01	0.00	2.37	0.62	<0.01	<0.01	<0.01	0.10	0.10	0.72
WK4	4.79	4.58	5.59	2.28	0.92				0.94	0.78	0.38
WK6	14.24	2.12	0.72	6.59	1.97				0.02	0.01	0.11
WK8	15.86	5.95	7.05	0.01	1.63				0.00	0.00	0.00

Ruminococcaceae_UCG-014											
WK2	1.46	0.00	1.87	11.37	1.75	0.58	0.21	0.02	0.73	0.92	0.03
WK4	6.74	5.53	6.60	6.73	0.84				0.66	0.96	1.00
WK6	7.38	7.57	1.81	5.25	1.05				0.95	0.06	0.45
WK8	4.84	4.79	5.03	0.23	0.96				0.99	0.94	0.10
Rikenellaceae_RC9_gut_group											
WK2	0.16	0.01	0.00	1.55	0.39	<0.01	<0.01	<0.01	0.89	0.889	0.23
WK4	5.99	9.87	3.46	1.31	1.72				0.43	0.61	0.35
WK6	4.45	5.91	0.00	8.75	1.25				0.64	0.16	0.18
WK8	21.59	3.15	3.62	0.00	2.30				0.00	0.00	0.00
Peptostreptococcus											
WK2	0.07	32.31	0.07	0.00	4.16	<0.01	<0.01	<0.01	0.00	1.00	0.99
WK4	0.00	0.00	0.00	1.39	0.35				1.00	1.00	0.18
WK6	0.00	0.00	0.00	0.00	0.00				1	1	1
WK8	0.00	0.00	0.00	10.41	1.45				1.00	1.00	1
[Ruminococcus]_gauvreauii_group											
WK2	1.54	0.02	0.27	1.60	0.39	0.47	<0.01	<0.01	0.18	0.26	0.95
WK4	4.76	2.44	4.07	6.70	0.87				0.36	0.78	0.44
WK6	0.36	4.33	5.44	1.76	0.68				0.01	0.00	0.32
WK8	0.00	0.49	0.46	0.78	0.23				0.49	0.52	0.28
Olsenella											
WK2	0.00	0.93	0.85	0.28	0.27	0.19	0.28	0.14	0.26	0.30	0.72
WK4	3.13	8.22	0.49	1.08	1.20				0.10	3.78	0.49

WK6	1.75	6.54	2.07	0.00	1.16				0.15	0.92	0.58
WK8	0.00	0.00	0.79	5.69	1.42				1.00	0.85	0.19
[Eubacterium]_coprostanoligenes _group											
WK2	0.00	0.01	0.00	0.70	0.17	0.67	0.29	0.62	0.99	1.00	0.17
WK4	1.08	2.61	3.51	0.65	0.60				0.37	0.17	0.80
WK6	0.49	0.57	0.00	3.75	0.47				0.93	0.58	0.00
WK8	1.13	2.02	2.33	0.00	0.28				0.10	0.03	0.04
norank_f__Lachnospiraceae											
WK2	3.11	0.53	13.31	0.32	3.22	0.43	0.27	0.53	0.78	0.29	0.77
WK4	0.00	0.72	0.00	0.00	0.14				0.08	1.00	1.00
WK6	0.00	2.99	1.92	0.00	0.60				0.08	0.25	1.00
WK8	0.00	0.37	0.28	1.11	0.17				0.40	0.52	0.02
Romboutsia						0.26	0.08	0.84			
WK2	0.00	0.00	0.00	2.30	0.44				1.00	1.00	0.06
WK4	0.00	4.63	4.61	5.96	1.61				0.35	0.35	0.23
WK6	0.00	0.00	0.84	2.49	0.46				1.00	0.49	0.06
WK8	0.00	2.37	1.93	0.00	0.74				0.30	0.39	1.00
Ruminococcaceae_UCG-013						0.01	0.1	0.49			
WK2	0.00	0.00	0.00	0.00	0.00				0.00	0.00	0.00
WK4	1.70	0.37	0.00	0.00	0.30				0.10	0.63	0.63
WK6	7.34	1.05	0.00	0.95	1.38				0.11	0.07	0.11
WK8	7.32	1.50	1.62	0.00	1.29				0.11	0.12	0.05
Fusobacterium						0.41	0.35	0.47			

WK2	0.00	14.40	0.77	0.00	3.57				0.18	0.94	1.00
WK4	0.00	0.00	0.00	0.00	0.00				1.00	1.00	1.00
WK6	0.00	0.00	0.00	0.00	0.00				1.00	1.00	1.00
WK8	0.00	0.00	0.00	0.00	0.00				1.00	1.00	1.00
[Ruminococcus]-torques-group											
WK2	1.81	8.39	1.39	1.50	2.08	0.43	0.31	0.36	0.30	0.95	0.96
WK4	0.00	0.00	0.00	0.00	0.00				1.00	1.00	1.00
WK6	0.00	1.33	6.48	0.00	1.61				0.78	0.19	1.00
WK8	0.00	0.00	0.00	1.01	0.14				1.00	1.00	0.00
Fungi											
Aspergillus											
WK2	1.49	45.93	13.75	37.66	6.60	0.39	0.01	0.91	0.01	0.43	0.03
WK4	54.17	65.28	20.32	50.05	5.05				0.21	0.00	0.63
WK6	44.27	47.72	30.49	74.38	6.82				0.84	0.44	0.11
WK8	52.00	62.77	59.47	26.36	4.47				0.20	0.37	0.01
Thermomyces											
WK2	0.00	18.06	5.03	12.48	4.92	0.38	0.61	0.18	0.24	0.74	0.41
WK4	1.18	0.67	4.77	4.04	0.80				0.81	0.11	0.20
WK6	0.32	18.51	0.96	0.27	3.73				0.10	0.95	0.10
WK8	5.53	0.01	0.03	16.78	2.82				0.44	0.45	0.13
Saccharomyces											
WK2	0.00^{b}	1.59^{b}	4.73^{b}	15.19^{a}	3.18	0.24	0.74	0.24	0.86	0.60	0.01
WK4	0.00	5.30	8.30	3.07	1.96				0.37	0.17	0.60
WK6	0.00	4.76	4.72	0.68	1.11				0.14	0.14	0.83

WK8	0.00	2.49	6.60	18.26	4.44				0.85	0.62	0.18
Melanocarpus											
WK2	0.00	0.21	2.53	16.11	2.94	0.90	0.51	0.57	0.98	0.74	0.05
WK4	1.71	0.00	11.76	2.51	1.54				0.60	0.01	0.80
WK6	0.96	2.68	0.10	0.18	0.43				0.13	0.44	0.48
WK8	2.54	0.09	0.01	20.04	2.79				0.67	0.66	0.01
Cutaneotrichosporon											
WK2	20.62	0.42	29.20	0.02	6.96	0.06	0.02	0.03	0.32	0.67	0.31
WK4	2.43	0.17	0.50	0.24	0.35				0.02	0.04	0.02
WK6	0.00	0.05	0.90	0.38	0.18				0.92	0.09	0.45
WK8	0.98	0.01	0.69	0.96	0.34				0.36	0.78	0.96
Pichia											
WK2	46.78	1.08	0.00	0.00	7.99	0.49	0.13	0.73	0.04	0.03	0.03
WK4	0.00	3.37	0.47	0.02	0.84				0.19	0.85	0.99
WK6	0.00	0.00	0.00	0.00	0.00				0.00	0.00	0.00
WK8	0.00	0.00	0.00	0.00	0.00				0.00	0.00	0.00
Wallemia											
WK2	0.00	2.10	3.21	4.74	0.92	0.28	0.78	0.89	0.44	0.21	0.08
WK4	0.55	2.46	5.21	1.00	0.64				0.19	0.01	0.75
WK6	0.29	1.51	4.42	2.18	0.90				0.64	0.16	0.47
WK8	2.36	1.51	1.54	0.51	0.31				0.33	0.35	0.05
Chrysosporium											
WK2	0.00	0.03	0.07	1.70	0.29	0.79	0.29	0.60	0.97	0.92	0.03
WK4	0.87	0.00	0.00	12.12	2.28				0.89	0.89	0.08

WK6	7.23	3.47	1.09	0.31	1.81				0.49	0.27	0.21
WK8	0.00	0.00	0.00	0.00	0.00				0.00	0.00	0.00
Acrostalagmus											
WK2	2.31	7.47	0.79	1.13	1.78	0.05	0.50	0.71	0.34	0.77	0.82
WK4	1.79	0.27	5.77	0.08	0.98				0.55	0.13	0.50
WK6	5.05	1.36	0.18	0.00	1.26				0.33	0.20	0.19
WK8	0.70	0.15	0.03	0.72	0.22				0.41	0.32	0.98
Microascus											
WK2	0.00	0.62	0.01	0.30	0.16	0.64	<0.01	0.68	0.20	0.99	0.54
WK4	0.00	0.38	0.00	0.49	0.09				0.09	1.00	0.03
WK6	3.69	0.07	0.33	1.73	0.70				0.08	0.10	0.32
WK8	2.89	5.26	2.67	0.33	0.77				0.26	0.92	0.22
Nigrospora											
WK2	0.00	0.47	0.47	0.90	0.21	0.04	0.23	0.20	0.45	0.45	0.16
WK4	0.00	0.00	0.00	0.00	0.00				0.00	0.00	0.00
WK6	3.27	0.97	0.97	0.24	0.81				0.35	0.35	0.23
WK8	0.00	0.00	0.00	0.00	0.00				0.00	0.00	0.00
Kazachstania											
WK2	0.00	1.80	0.00	0.00	0.31	0.71	0.11	0.29	0.03	1.00	1.00
WK4	0.04	0.00	0.00	0.29	0.07				0.84	0.84	0.26
WK6	0.00	0.00	0.47	0.00	0.82				1.00	0.04	1.00
WK8	0.00	0.00	0.00	0.00	0.00				0.00	0.00	0.00
unclassified_c__Sordariomycetes											
WK2	0.00	1.40	3.32	2.20	0.78	0.45	0.26	0.76	0.55	0.17	0.35

WK4	1.01	10.96	3.05	6.63	1.90				0.08	0.70	0.30
WK6	10.48	1.60	2.64	2.88	1.47				0.03	0.05	0.06
WK8	4.61	2.94	2.86	1.52	0.84				0.52	0.50	0.24
unclassified_f__Plectosphaerellaceae											
WK2	0.06	0.23	3.17	0.78	0.61	0.65	0.23	0.31	0.92	0.08	0.67
WK4	0.17	2.09	0.00	2.31	0.54				0.22	0.91	0.18
WK6	0.77	0.83	0.15	1.21	0.31				0.95	0.52	0.65
WK8	9.34	4.27	5.10	0.20	1.72				0.31	0.39	0.08
unclassified_k__Fungi											
WK2	0.25	3.25	0.38	0.93	1.02	0.80	0.02	0.63	＜0.01	＜0.01	＜0.01
WK4	2.68	0.06	0.00	0.00	0.58				0.12	0.11	0.11
WK6	0.48	0.70	22.05	0.21	5.43				0.99	0.19	0.99
WK8	1.30	0.86	0.43	0.34	0.24				0.53	0.24	0.20
unclassified_f__Sporormiaceae											
WK2	0.00	0.00	0.00	0.00	0.00	0.29	0.85	0.91	1	1	1
WK4	9.51	0.23	0.41	2.22	1.63				0.05	0.05	0.10
WK6	0.00	0.00	0.00	0.00	0.00				1	1	1
WK8	3.39	0.83	1.22	0.01	0.85				0.33	0.40	0.20

[a-b] The values in the same row with different superscripts are significantly different ($P < 0.05$), while values with the same or no superscripts mean no significant difference ($P ＞ 0.05$).

[1]Treatments: C = no supplementation (control); T1 = 0.5 g/calf/day; T2 =1 g/calf/day; T3 = 2 g/calf/day.

[2]SEM = Standard error of mean.

T
